# Supplementary material for: Identification of Freezing-Responsive microRNAs and Their Targets in Chinese Jujube by Small RNA and Degradome Sequencing
Source: Int J Mol Sci. 2026 Sep 4;27(17):7912. doi: 10.3390/ijms27177912 (PMC13566890; doi:10.3390/ijms27177912)
Supplement: Supplementary file 1 [file ijms-27-07912-s001.zip › Supplementary Material S2/novel_miR_83.pdf]

Provisional ID	:	novel_miR_83
Score total	:	107
Score for star read(s)	:	3.9
Score for read counts	:	97
Score for mfe	:	4.5
Score for randfold	:	1.6
Score for cons. seed	:	
Total read count	:	202
Mature read count	:	90
Loop read count	:	0
Star read count	:	112

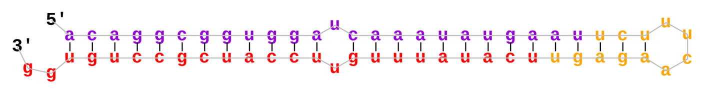

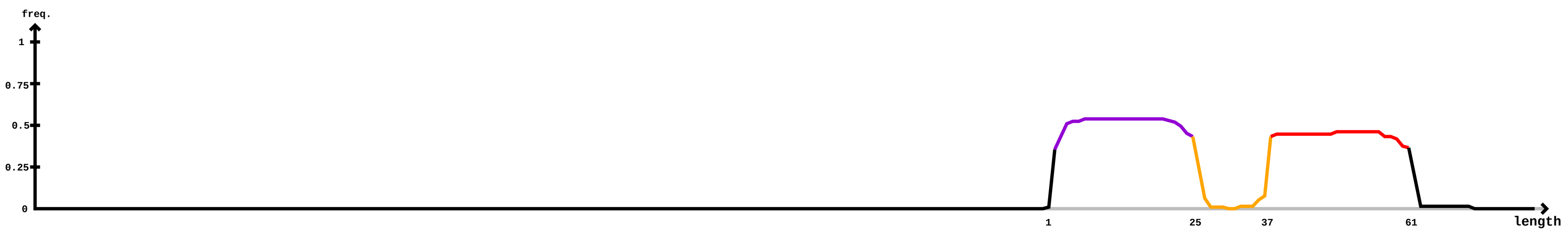

Star **Mature**

```
5'- cuaugagaagacagaugaaaaauucaucugucaugugaagacugccuaugcauacaaagaauuccaucaaaauacuaagcgaugggcuuguauagcauuugcagccuuugucaaacucagaagaaaaugcauuuuuuugcacuuucuucaaagguugcuuccgcauacacaggcgguggaucaaauaugaauucuuucaagaguucauauuuguuccaucgccugugg augcucaagcagcuuauaauga -3'  obs
    cuaugagaagacagaugaaaaauucaucugucaugugaagacugccuaugcauacaaagaauuccaucaaaauacuaagcgaugggcuuguauagcauuugcagccuuugucaaacucagaagaaaaugcauuuuuuugcacuuucuucaaagguugcuuccgcauacacaggcgguggaucaaauaugaauucuuucaagaguucauauuuguuccaucgccuguggaugcucaagcagcuuauaauga      exp
    ...((((..((((((((((...))))))))))).(((((((.((((..((((((((((.(...((((((...........)))))))))))))).))))..)))).)))))).))..))))(((((((.((((.....)))).)))))))..((((((((..(((..(((((((((((((.(((((((((((((....))))))))))))).)))))))))))))..)))..)))))))))......
```

read	reads	mm	sample
cacaggcgguggaucaaauaugaa	1	0	JCK-1
cacaggcgguggaucaaauaugaa	1	0	JCK-2
acaggcgguggaucaaaua	1	0	JCK-3
acaggcgguggaucaaaua	1	0	LT-3
acaggcgguggaucaaauaug	1	0	JCK-1
acaggcgguggaucaaauaug	1	0	JCK-3
acaggcgguggaucaaauauga	4	0	JCK-2
acaggcgguggaucaaauauga	2	0	LT-3
acaggcgguggaucaaauauga	2	0	JCK-3
acaggcgguggaucaaauaugaa	2	0	LCK-3
acaggcgguggaucaaauaugaau	23	0	JCK-3
acaggcgguggaucaaauaugaau	25	0	JCK-2
acaggcgguggaucaaauaugaau	10	0	JCK-1
caggcgguggaucaaauau	2	0	JCK-2
cCggcgguggaucaaauaugaauu	1	1	JCK-2
caggcgguggaucaaauaugaauu	4	0	JCK-1
caggcgguggaucaaauaugaauu	2	0	JCK-3
caggcgguggaucaaauaugaauu	7	0	JCK-2
aggcgguggaucaaauaug	3	0	JCK-2
aggcgguggaucaaauauga	1	0	JCK-2
aggcgguggaucaaauaugaauu	1	0	JCK-3
aggcgguggaucaaauaugaauu	1	0	JCK-2
aggcgguggaucaaauaugaauuc	3	0	JCK-2
aggcgguggaucaaauaugaauuc	3	0	JCK-3
aggcgguggaucaaauaugaauuc	4	0	JCK-1
ggcgguggaucaaauaugaau	2	0	JCK-2
ggcgguggaucaaauaugaau	1	0	JCK-1
cgguggaucaaauaugaauuc	1	0	LT-2
cgguggaucaaauaugaauucuuu	2	0	JCK-2
agaguucauauuuguuccaucgcc	1	0	JCK-3
agaguucauauuuguuccaucgcc	1	0	JCK-2
agaguucauauuuguuccaucgcc	1	0	LT-1
guucauauuuguuccaucgcc	2	0	JCK-1

```
Star                                                    Mature
cuaugagaagacagaugaaaaauucaucugucaugugaagacugccuaugcauacaaagaauuccaucaaaauacuaagcgaugggcuuguauagcauuugcagccuuugucaaacucagaagaaaaugcauuuuuuugcacuuucuucaaagguugcuuccgcauacacaggcgguggaucaaauaugaauucuuucaagaguucauauuuguuccaucgccuguggaugcucaagcagcuuauaauga
..........................................................................................................................................................................................................guucauauuuguuccaucgcc...........................   1    0    JCK-2
..........................................................................................................................................................................................................guucauauuuguuccaucgccugu........................   2    0    JCK-1
..........................................................................................................................................................................................................guucauauuuguuccaucgccugu........................   2    0    JCK-3
..........................................................................................................................................................................................................guucauauuuguuccaucgccugu........................   1    0    JCK-2
...........................................................................................................................................................................................................uucauauuuguuccaucgccugu........................   1    0    JCK-2
...........................................................................................................................................................................................................uucauauuuguuccaucgccugu........................   2    0    JCK-1
...........................................................................................................................................................................................................uucauauuuguuccaucgccugug.......................   2    0    JCK-1
............................................................................................................................................................................................................ucauauuuguuccaucgccug.........................   1    0    JCK-2
............................................................................................................................................................................................................ucauauuuguuccaucgccug.........................   2    0    JCK-1
............................................................................................................................................................................................................ucauauuuguuccaucgccugu........................   1    0    JCK-1
............................................................................................................................................................................................................ucauauuuguuccaucgccugugg......................   17   0    JCK-1
............................................................................................................................................................................................................ucauauuuguuccaucgccugugU......................   1    1    JCK-3
............................................................................................................................................................................................................ucauauuuguuccaucgccugugg......................   18   0    JCK-3
............................................................................................................................................................................................................ucauauuuguuccaucgcAugugg......................   2    1    JCK-2
............................................................................................................................................................................................................uNauauuuguuccaucgccugugg......................   1    1    JCK-3
............................................................................................................................................................................................................ucauauuuguuccaucgccugugU......................   1    1    JCK-1
............................................................................................................................................................................................................ucauauuuguuccaucgccugugg......................   29   0    JCK-2
............................................................................................................................................................................................................ucauauuuguuccaucgccugugU......................   1    1    JCK-2
.............................................................................................................................................................................................................cauauuuguuccaucgccugugg......................   1    0    JCK-1
.............................................................................................................................................................................................................cauauuuguuccaucgccugugg......................   2    0    JCK-3
.......................................................................................................................................................................................................................ccaucgccuguggaugcucaagc............   1    0    JCK-2
.......................................................................................................................................................................................................................ccaucgccuguggaugcucaagc............   2    0    JCK-1
```
